# Nearing the Finish Line: Steady Progress in the Development of Complement Inhibitors for Glomerular Disease

**DOI:** 10.1016/j.ekir.2024.11.1370

**Published:** 2024-12-10

**Authors:** Joshua M. Thurman

**Affiliations:** 1 Department of Medicine, School of Medicine, Anschutz Medical Campus, University of Colorado, Aurora, Colorado, USA

See Clinical Research on Page 432

The complement system is activated in most, if not all, forms of glomerulonephritis. In immune-complex–mediated diseases, glomerular IgG and IgM deposits trigger activation of the classical pathway (Figure 1). In several other glomerular diseases, uncontrolled activation of the alternative pathway is the primary driver of glomerular injury.^1^ In C3 glomerulopathy (C3G), for example, immunofluorescence microscopy reveals glomerular C3 deposits that are out of proportion to the Ig. These diagnostic criteria—bright C3 with a relative paucity of glomerular Ig—provide a clue that activation does not involve the classical pathway, and preclinical models have convincingly shown that glomerular complement activation in this disease principally involves the alternative pathway.^2^

**Figure 1:**
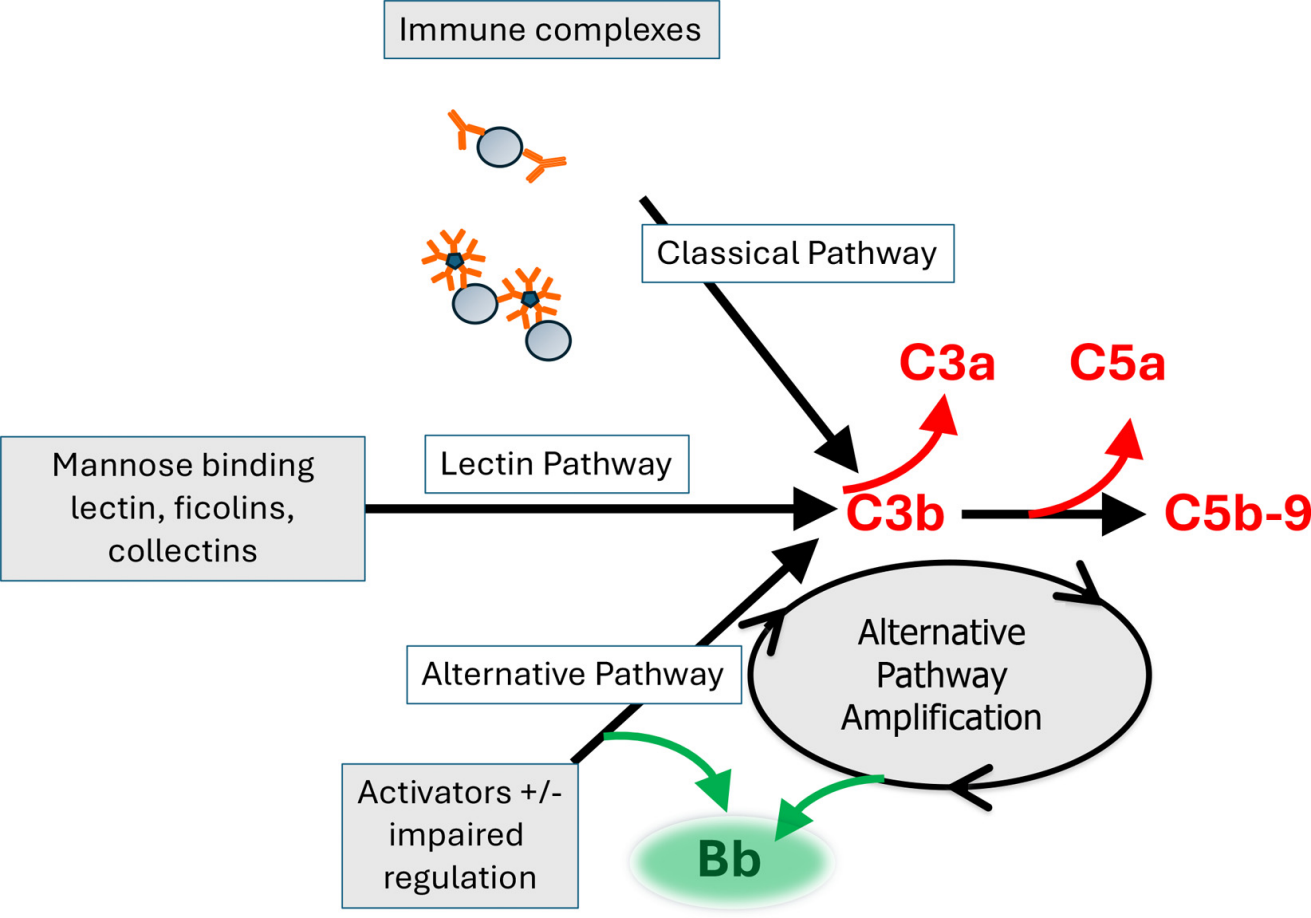
Complement activation in glomerulonephritis. Activation through the classical, lectin, and alternative pathways of complement generates C3b, which is covalently fixed to glomerular surfaces. Regardless of how C3b is initially generated, complement activation is subsequently amplified through the alternative pathway. Complement activation generates several proinflammatory fragments, including C3a, C3b, C5a, and C5b-9. C5b-9 can activate target cells, and soluble C5b-9 (sC5b-9) in plasma and urine can also serve as a biomarker of complement activation. Activation of the alternative pathway generates soluble Bb, a protein that can be measured as a biomarker of alternative pathway activation.

Activation of the complement cascade through any of the initiation pathways generates the same downstream proinflammatory molecules, namely C3a, C3b, C5a, and C5b (which nucleates formation of C5b-9). Each of these, in turn, has potent effects and may contribute to tissue injury. Although IgG, itself, can mediate glomerulonephritis through noncomplement mechanisms,^3^ it is noteworthy that C3G may have the worst prognosis among the primary glomerular diseases. One can infer, therefore, that complement activation is a key driver of glomerular injury, with or without Ig.

Although there is a strong rationale for using complement inhibitory drugs to treat both immune-complex–mediated and nonimmune-complex–mediated glomerular diseases, the treatment strategy may differ in these 2 settings. In immune-complex glomerular diseases, drugs that target the adaptive immune response and reduce production of autoantibodies should eventually reduce classical pathway activation by Ig in the glomerulus. In these diseases, therapeutic complement inhibitors will still be useful for rapidly suppressing glomerular inflammation while waiting for existing immune complexes to be cleared. Furthermore, the clinical response to cytotoxic drugs is incomplete in most clinical trials, so reliable methods of blocking glomerular complement activation can provide renoprotection to patients who do not respond to other therapies.

For “pure” complement-mediated diseases, such as C3G, complement inhibitors block the primary disease process and should be sufficient to effectively cure the disease. Interestingly, even though glomerular IgG deposits are scant in C3G, autoreactive IgG or IgG fragments may play a critical role in the activation of the alternative pathway. “Nephritic factors” are autoantibodies that disrupt regulation of the alternative pathway, and they are frequently detected in serum from patients with C3G.^3^ C3G in adult patients is also frequently associated with monoclonal gammopathies, and the paraproteins may function as nephritic factors. It is possible, therefore, that immunosuppressive drugs will have an important role in the treatment of C3G, even if complement inhibitors prove effective.

As treatments for C3G enter the clinic, another important consideration will be the duration of treatment. Although acquired and congenital defects in complement regulation may put patients at life-long risk of relapse, C3G is still a disease characterized by flares and remissions. Indefinite treatment with complement inhibitory drugs is, therefore, probably unnecessary, and it is important that we develop accurate tests or biomarkers for distinguishing patients with active disease from those who are in remission. Plasma C3 and C4 levels have traditionally been used to monitor complement activation; however, these tests are neither sensitive nor specific enough to track complement activation in the kidney. In fact, C3 levels are only reduced in approximately 50% of adult patients with C3G.^4^ Other complement biomarkers, such as the activation fragments, may be better indicators of ongoing complement activation.^5^

Several new anticomplement drugs have been tested or in recent years. Eculizumab, a monoclonal antibody to C5, was the first of these drugs approved by the US Food and Drug Administration for treatment of kidney disease. Unfortunately, the results of using eculizumab to treat C3G have been disappointing.^4^ This may reflect the challenges in studying a rare and heterogeneous disease; however, it might also indicate that it is necessary to block generation of the upstream complement activation fragments C3a and C3b. This can be achieved by targeting complement activation at the level or C3, or by inhibiting the specific activation pathway. In this issue of KI Reports, Nester and colleagues report the results from the extension phase of a phase 2 clinical trial in which patients with C3G were treated with iptacopan,^6^ a drug that blocks activation of factor B and selectively prevents complement activation through the alternative pathway. The mechanism of action for this drug is well-suited to alternative pathway–mediated diseases, such as C3G. It is also an oral medication, reducing the burden placed on patients who need prolonged treatment.

Investigators had previously reported the interim results for this study.^7^ Twenty-seven patients were originally enrolled for treatment with iptacopan: 16 patients had native kidney disease, and 11 were kidney transplant recipients who had recurrent disease in their allografts. After 12 weeks of treatment, proteinuria was reduced by approximately 45% in patients with native kidney disease. Baseline proteinuria levels were too low in kidney transplant recipients to assess the efficacy of treatment; however, immunofluorescence analysis of biopsies obtained before and after treatment demonstrated that iptacopan reduced the intensity of the glomerular C3 deposits.

In the current report, the authors present follow-up data for 26 of the original patients (16 native kidney and 10 KTRs) who consented to 9 months of additional treatment in an open label extension phase. In patients with native kidney disease, 53.3% of the patients reached a composite kidney end point, which comprised stabilization of the estimated glomerular filtration rate, a reduction in proteinuria, and an increase in plasma C3 levels. The mean values for each of these clinical end points were improved, and C3 levels normalized in 8 of the 16 patients. The authors also measured plasma sC5b-9 and Bb, fragments generated during alternative pathway activation (Figure 1). Treatment was associated with reduced levels of these complement biomarkers. This follow-up study again includes analysis of C3 deposition in KTR biopsies, 4 of which were collected after 12 months of treatment. C3 deposits were reduced in 3 of the 4 patients for whom biopsies were performed. Regarded together, these new data provide additional evidence that iptacopan blocks alternative pathway activation in patients with C3G and reduces C3 deposition in glomeruli.

Several aspects of this study are worth highlighting. Biomarkers of alternative pathway activation were reduced, but not normalized, in treated patients. There was also heterogeneity among the patients in their response to treatment. These observations suggest that alternative pathway inhibition by iptacopan may have been incomplete, and it is possible that a greater degree of complement inhibition would be more effective. Another interpretation of these results, however, is that the complement system does not need to be completely blocked to suppress glomerular inflammation. Pegcetacoplan is a C3 inhibitor that also appears to be effective in C3G,^8^ and a comparative analysis of the 2 drugs may inform these issues. Another interesting aspect of the study is that 7 of the patients with native kidney disease were maintained on mycophenolate derivatives, as were the KTRs. Complement inhibition in this disease, therefore, is additive to the beneficial effects of standard immunosuppression. One limitation of the study is that it relies on surrogate end points for kidney protection, and longer-term follow-up will be necessary to confirm the efficacy of the drug. Comparison of the decline in estimated glomerular filtration rate before and after starting iptacopan, however, showed that the treatment was associated with a slowing in the decline in estimated glomerular filtration rate. The authors also reported that treatment was associated with a reduction in levels of urine neutrophil gelatinase–associated lipocalin, a marker of tubular injury.

The advent of therapeutic complement inhibitors is an important advance for the field of nephrology. Complement inhibitors enable clinicians to block a critical part of the immune system that is not directly targeted by glucocorticoids or cytotoxic drugs. These agents will be useful adjuncts for treatment of immune-complex diseases, and they will likely become a cornerstone of treatment for C3G. Among the complement inhibitors, iptacopan is advantageous for several reasons. It blocks alternative pathway activation while leaving the other complement pathways intact. This may reduce the pathologic effects of complement activation in the kidney, without impeding its other physiologic and protective functions. Several studies have now shown that iptacopan is safe and well-tolerated in patients with kidney disease. Importantly, the results of a phase 3 study of iptacopan in IgA nephropathy were also recently reported^9^ and the drug has received accelerated approval from the US Food and Drug Administration for this indication.

Treatment with iptacopan led to rapid reductions of proteinuria in patients with C3G or IgA nephropathy;^6^^,^^9^ thus providing compelling evidence in support of alternative pathway inhibition as an effective treatment for alternative pathway–mediated glomerular diseases. Additional clinical trials are currently testing the drug in antineutrophil cytoplasmic antibody–associated vasculitis (NCT06388941) and atypical hemolytic uremic syndrome (NCT04889430). There is also a clinical trial in immune complex–mediated membranoproliferative glomerulonephritis (NCT05755386), a disease in which activation is probably initiated through the classical pathway. These trials should help to establish whether the rapid beneficial effects of alternative pathway inhibition in IgA nephropathy and C3G can be generalized to glomerulonephritis of other etiologies.

The successful use of complement inhibitory drugs in multiple types of glomerulonephritis puts us on the cusp of having effective treatment options for previously untreatable diseases. Furthermore, complement inhibitors may reduce the need for glucocorticoids in diseases for which effective immunosuppressive treatments already exist.S1 Finally, the investigators of the current study should be commended for exploring new complement biomarkers. Validation of these tests may help clinicians to monitor immunologic activity within the glomerulus, and to personalize each patient’s treatment. Better tools for assessing glomerular disease activity will benefit patients with all forms of autoimmune and autoinflammatory glomerulonephritis, even those who are not treated with complement inhibitory drugs.

## Disclosure

JMT is a consultant for Q32 Bio, Inc., a company developing complement inhibitors. He holds stock in and will receive royalty income from Q32 Bio, Inc. JMT holds stock in Compsit3, Inc.

## Supplementary Material

Supplementary File (PDF) **Supplementary References**.

## Footnotes

Supplementary File (PDF)

**Supplementary References**
