# Supplementary material for: Nearing the Finish Line: Steady Progress in the Development of Complement Inhibitors for Glomerular Disease
Source: Kidney Int Rep. 2024 Dec 10;10(2):302–5. doi: 10.1016/j.ekir.2024.11.1370 (PMC11843313; doi:10.1016/j.ekir.2024.11.1370)
Supplement: Supplementary File (PDF) — Supplementary References. [file mmc1.pdf]

Supplementary Reference

S1. Jayne DRW, Merkel PA, Schall TJ, Bekker P; ADVOCATE Study Group. Avacopan for the treatment of ANCA-associated vasculitis. *N Engl J Med*. 2021;384**:**599–609. doi:10.1056/NEJMoa2023386
